# Mass Spectrometric Characterization of Narcolepsy-Associated Pandemic 2009 Influenza Vaccines

**DOI:** 10.3390/vaccines8040630

**Published:** 2020-10-30

**Authors:** Aditya Ambati, Guo Luo, Elora Pradhan, Jacob Louis, Ling Lin, Ryan D. Leib, Hanna Maria Ollila, Thomas Poiret, Christopher Adams, Emmanuel Mignot

**Affiliations:** 1 Stanford Center for Sleep Sciences and Medicine, Department of Psychiatry and Behavioral Sciences, Stanford University, 3165 Porter Drive, Stanford, CA 94304, USA; ambati@stanford.edu (A.A.); gluo@stanford.edu (G.L.); elora.pradhan@gmail.com (E.P.); louis.jacob.contacts@gmail.com (J.L.); linglin2058@gmail.com (L.L.); hanna.m.ollila@helsinki.fi (H.M.O.); 2 Stanford Mass Spectrometry Core, 333 Campus Drive, Mudd 175, Stanford University, Stanford, CA 94305, USA; rdleib@stanford.edu (R.D.L.); Christopher.Adams@bruker.com (C.A.); 3 Department of Laboratory Medicine, Karolinska Institutet, 14152 Stockholm, Sweden; thomas.poiret@ki.se

**Keywords:** narcolepsy, influenza vaccine, mass spectrometry, mutations

## Abstract

The onset of narcolepsy, an irreversible sleep disorder, has been associated with 2009 influenza pandemic (pH1N1) infections in China, and with ASO3-adjuvanted pH1N1 vaccinations using Pandemrix in Europe. Intriguingly, however, the increased incidence was only observed following vaccination with Pandemrix but not Arepanrix in Canada. In this study, the mutational burden of actual vaccine lots of Pandemrix (n = 6) and Arepanrix (n = 5) sourced from Canada, and Northern Europe were characterized by mass spectrometry. The four most abundant influenza proteins across both vaccines were nucleoprotein NP, hemagglutinin HA, matrix protein M1, with the exception that Pandemrix harbored a significantly increased proportion of neuraminidase NA (7.5%) as compared to Arepanrix (2.6%). Most significantly, 17 motifs in HA, NP, and M1 harbored mutations, which significantly differed in Pandemrix versus Arepanrix. Among these, a 6-fold higher deamidation of HA146 (p.Asn146Asp) in Arepanrix was found relative to Pandemrix, while NP257 (p.Thr257Ala) and NP424 (p.Thr424Ile) were increased in Pandemrix. DQ0602 binding and tetramer analysis with mutated epitopes were conducted in Pandemrix-vaccinated cases versus controls but were unremarkable. Pandemrix harbored lower mutational burden than Arepanrix, indicating higher similarity to wild-type 2009 pH1N1, which could explain differences in narcolepsy susceptibility amongst the vaccines.

## 1. Introduction

Type 1 Narcolepsy (T1N) is a disabling disorder characterized by excessive daytime sleepiness, irresistible daytime sleep attacks, and sudden episodes of loss of muscle tone following emotions such as laughter, a symptom known as cataplexy [1]. Genetic and immunological studies have shown that the disorder is autoimmune, and likely mediated by T cell attacks targeting hypocretin producing neurons, a population of 20,000 neurons located in the posterior hypothalamus [2,3]. Hypocretins are critical regulators of wakefulness and Rapid Eye Movement sleep (REM sleep), and lack of hypocretin transmission is causal to the symptoms of the disorder [4,5]. 

Until recently, the rationale for an autoimmune basis for narcolepsy was based mainly on epidemiological and genetic evidence. First, there is a uniquely strong association between narcolepsy and 6p21.3, a region of the genome, including the human leukocyte antigen HLA locus, more specifically, 97% of narcoleptic patients carry at least one copy of HLA DQB1*06:02 (DQ0602) across ethnicities, an HLA class II allele found in 25% of the general population [6,7,8]. Additional weak effects in HLA-A*11:01 [9] and an impact of DQB1*03:01 on the age of onset were also observed [10]. Genome-wide association studies have found that narcolepsy is associated with T-cell receptor loci TRA & TRB, and immune genes such as CTSH, P2RY11, ZNF265, IFNAR1, and TNSF4 [10,11,12]. As all these loci are involved in immune regulation and other autoimmune diseases, an autoimmune mediation of hypocretin cell death has long been proposed as the cause of narcolepsy. Of notable interest is the fact the TCR loci associated with narcolepsy are modulators of TRAJ24 and TRBV4-2, TCR segments only involved in 0.8% and 0.7% of the total TCR repertoire, respectively.

Narcolepsy studies have described environmental triggers in addition to the genetic susceptibilities; specifically, while some studies have noted increased humoral (IgG) and cellular (IFNγ) responses to streptococcus pyogenes infection [13,14] others have reported no differences in Chinese narcolepsy patients [15]. Similarly, epidemiological studies have suggested increased frequency of strep infections and flu-like illness in patients before developing narcolepsy [16]. Most recently, the data has most strongly implicated influenza-A infection and vaccination. Following the 2009–2010 H1N1 “swine flu” influenza pandemic infection in China, increased T1N onsets were observed [10]. In European countries, a significant 4–16-fold increase in the risk of developing narcolepsy in children was observed a few months following an aggressive pandemic H1N1 (pH1N1) flu vaccination campaign with the vaccine Pandemrix [17,18,19,20,21,22,23,24]. In these cases, Pandemrix increased the incidence of narcolepsy by a factor of 2–15, from around 1/150,000 to 1/15,000 cases per year in children. The risk was mostly increased in younger children, but there was still a significant, albeit weaker effect in adults [20]. Similar increases in narcolepsy incidence were not observed in countries where other pandemic vaccines, notably Arepanrix in Canada, were used, thereby elucidating the impression that Pandemrix uniquely triggered narcolepsy [25].

Starting in May 2009 in response to the pandemic H1N1, vaccine manufacturers began to plan the production of a specialized vaccine targeting the new strain for vaccination the following winter, a concise timeline. The creation of vaccine strains involves growing strains derived from pathogenic strains reassorted with PR8 (an old 1918-H1N1-like strain 08/35 from Puerto Rico) in eggs. The reassortant strain is typically constructed by the New York Medical Center (NYMC), which is then distributed to manufacturers for growing millions of doses in eggs in specialized factories. The NYMC H1N1-like vaccine strains produced for the 2009–2010 swine flu campaign used A/H1N1/California/7/2009 as the pathogenic strain, so that only Hemagglutinin (HA), Neuraminidase (NA), and polymerase PB1 are derived from A/H1N1/California/7/2009, while other proteins are PR8 derived. In close succession, NYMC-X-179A and NYMC X-181, a higher growth reassortant derived from X-179A were created, with the former strain having been used more widely (X-181 was only used toward the end of the season in some cases) [26]. Once distributed, vaccine manufacturers used their own patented process to produce vaccines using X-179A and X-181. In egg-based vaccine production processes, candidate vaccine viruses are grown in eggs per current FDA regulatory requirements. To do so, X-179A is injected into fertilized hen’s eggs and incubated for several days to allow viruses to replicate. The virus-containing allantoic fluid is then harvested from the eggs, viruses inactivated (killed), and virus antigens purified, with the general goal of preferentially isolating viral surface proteins HA and NA, which are most important for protective antibody responses sought with vaccine administration [27,28].

Using this process, the manufacturer GlaxoSmithKline created Pandemrix and Arepanrix, both AS03-adjuvanted vaccines; the AS03 adjuvant is an immunological agent added to the vaccine to boost the immune system’s response to the target antigen while reducing the dosage of the viral antigen (antigen sparing, a property that was desirable considering short time of production) [28]. Arepanrix was produced and used in Canada around the same time that Pandemrix was deployed in Europe, but it did not sharply increase narcolepsy risk in Quebec [25,29]. This is notable as Arepanrix is almost identical to Pandemrix with a same adjuvant from the same geographic origin as well as similar viral composition. However, different viral antigen purification techniques were used for either vaccine—Fluarix for Pandemrix and Flulaval for Arepanrix [30]. Why Pandemrix in Europe and not Arepanrix in Canada triggered narcolepsy cases is unknown. One possibility may be the differential composition of the vaccine, notably due to the fact the viral antigens were extracted using different manufacturing processes. Another possibility involves the presence of other factors differentiating Canada and Europe at the time of vaccination. After all, it is essential to note that even with Pandemrix, only 1/16,000 vaccinated children (or 1/4000 DQB0602 positive subjects) developed narcolepsy [19], so that almost undoubtedly other environmental or stochastic factors are involved in addition to vaccine trigger and genetic background. As Canadian and northern European populations are similar in term of DQ0602 frequency (and other narcolepsy-associated genetic factors), a possibility could be a differential immune history of both population regarding past flu or the fact that in Northern Europe vaccination occurred shortly before or exactly when the pandemic H1N1 infection affected the population. At the same time, in Canada the bulk of vaccination occurred immediately after the pandemic flu started to change the population [23].

Our understanding of narcolepsy immunology changed significantly a few months ago, thanks to two studies [31,32]. Latorre et al. used an ultrasensitive technique involving polyclonal expansion and cloning of CD45RA^−^CD4^+^ memory T cells, found strong line reactivity to HCRT in all patients versus no or limited responses in 12 DQ0602 controls, with significantly higher reactivity in T1N. These authors found responses to seasonal influenza vaccine to be comparable in patients and controls, concluding that flu antigen mimicry could not be detected [31]. On other hand, Luo et al. [32] screened peptides derived from HCRT and flu strains including pH1N1 for DQ0602 binding and presence of cognate tetramer-peptide specific CD4^+^ T cells in 35 T1N cases and 22 DQ0602 controls finding higher reactivity to influenza pHA_273–287_ (pH1N1 specific) and C-amidated but not native version of HCRT_54–66_ and HCRT_86–97_ sequences (two homologous sequences we denoted HCRT_NH2_) in T1N when presented by DQ0602. 

Relatively few studies have examined composition differences across flu vaccines such as Pandemrix and Arepanrix. Comparative studies of antibody reactivity to Pandemrix and Arepanrix antigens in both post-Pandemrix patients and control individuals found that post-Pandemrix-vaccinated children had poorer antibody reactivity to Arepanrix, suggesting antigenic differences in antibody determinants [33]. This could be important, although one would expect that differential susceptibility would be more likely due to T cell response differences. High-resolution gel electrophoresis quantitation and Mass Spectrometry (MS) identification analyses revealed higher amounts of structurally altered viral nucleoprotein (NP) in Pandemrix versus Arepanrix, a finding that can also be noted in a 2-D gel study by Jacob et al. [30]. These results suggest complex protein aggregate conformation differences that could be relevant to differential activity of these vaccines, most notably in terms of antibody response to NP. Jacob et al. [30] comparing single batches of Pandemrix and Arepanrix found that viral proteins NP and NA as well as selected non-viral chicken proteins (PDCD6IP, TSPAN8, H-FABP, HSP, and TUB) were more abundant in Pandemrix compared to Arepanrix. The study also found an accumulation of a specific mutation in Arepanrix, 146N to D in Arepanrix, but as the batch studied had only been synthesized in 2010 and had never been used for vaccination, it was hard to be sure if this mutation had been present in earlier batches. Finally, Ahmed et al. [34] found an increased quantity of cross-reactive antibodies to structurally altered NP epitope NP116I with an epitope from HCRTR2 protein in narcolepsy cases. Although the proportion of NP116I mutations in both Arepanrix and Pandemrix was similar and, therefore, unlikely to be a causal effect, increased cross-reactive antibodies between NP116I and HCRTR2 were not observed in other studies [35].

In the current study, we extended the previous findings from Jacob et al. [30] by characterizing the protein content and mutational burden in 6 Pandemrix lots and 5 Arepanrix lots in relation to X-179A, the influenza strain from which both vaccines were derived. Data shows batch diversity in mutation burden that could be relevant to vaccine responses in some cases but not a distinct difference that could explain why Pandemrix was more associated with narcolepsy onsets than Arepanrix. Mutational drift and low-level mutations are present in some vaccine batches (as occurring in wild type virus), and this should be taken into consideration when studying the effects of these vaccines in the population.

## 2. Methods

### 2.1. Vaccines 

Pandemrix doses used in the 2009 vaccination campaign were sourced from Sweden (3), France (1), and additionally provided by GSK (2), while Arepanrix was sourced from Canada (4) and supplied by GSK (1). All except for two lots were monovalent bulks (see Table 1). The vaccines were all derived from the X-179A, a vaccine strain that built on the PR8 backbone and was populated with pH1N1 proteins (HA, NA and PB1) from the A/California/07/2009 strain. Pandemrix lots were prepared using the Fluarix process in Dresden while the Arepanrix lots were processed using the Flulaval process in Saint Foye as described [30]. All the vaccines were stored at +4C until assayed.

### 2.2. Mass Spectrometry (MS)

MS was performed on Trypsin/Lys-C (Promega Corporation, Madison, WI, USA), and Chymotrypsin (Promega) digests of Pandemrix and Arepanrix samples (6.5 µg each) and detailed elsewhere [30]. Raw mass spectra of each vaccine digest (trypsin and chymotrypsin separately) were analyzed using a combination of Preview and Byonic v.1.4 spectral analysis software (Protein Metrics Inc., Cupertino, CA, USA). Complementary approaches were pursued. Spectrograms derived from each vaccine digest were mapped to (i) a concatenated FASTA file containing the canonical proteomes for five influenza viral strains: A/California/07/2009 (H1N1), NYMC X-181A (identical to NYMC X-181), NYMC X-179, NYMC X-179A, and A/Puerto Rico/8/1934, and (ii) an in-silico mutant peptide library artificially generated from X-179A that included all possible single amino acid substitutions in the five most frequent flu proteins out of 14,894 unique proteins. Additional validation was done using typical False Discovery Rate (FDR) by including common contaminants and sequence reverses in the FASTA database for all searches. Data was qualified down to a 1% FDR level for proteins. Further fragments that passed a threshold of log probability of 2 and were present four or more times in at least one vaccine digest were considered for further analysis.

### 2.3. Statistical Analysis 

After the MS vaccine, digest files were aligned to the reference library, and the mass spectra were exported as CSV files from Byonic v.1.4 spectral analysis software (Protein Metrics, CA, USA) and then processed through custom R and Python scripts. Using the Biopython library, the protein sequences for the five reference flu proteins used in the MS processing were retrieved. For each reference flu protein, all corresponding peptides were extracted from the MS database and compared to the reference protein sequence at each motif. From this comparison, the frequency of mutated and wild-type amino acids relative to the X-179A derived viral strain was characterized at each position in each protein in each vaccine batch. Then, mutation proportions were computed for each position. After trypsin and chymotrypsin data from the same vaccine batch were merged, a two-tailed Student’s t-test was used to determine significant mutation proportions between Pandemrix and Arepanrix batches, thus pinpointing the specific mutations that significantly distinguished Arepanrix and Pandemrix vaccines.

### 2.4. DQ0602 Binding

The in-vitro binding has been described in detail in Luo et al. [32,35] and is briefly described here, Peptide competing binding assays were conducted by incubation of 25 nM DQ0602, 100 nM HLA-DM, 1 μM biotin-conjugated Epstein-Barr virus (EBV) epitope (Bio-EBV, Bio-GGGRALLARSHVERTTDE), with 40 μM of the competitor peptide in reaction buffer (100 mM acetate, pH = 4.6, 150 mM NaCl, 1% BSA, 0.5% Nonidet P-40 (IGEPAL CA-630, Sigma, St. Louis, MO, USA), 0.1% NaN3) in duplicate for 3 days at 37 °C. The reaction was quenched by adding two volumes of neutralization buffer (100 mM Tris-HCl, pH = 8.6, 150 mM NaCl, 1% BSA, 0.5 Nonidet P-40 (IGEPAL CA-630, Sigma), 0.1% NaN3). Monoclonal anti-DQ (SPV-L3) antibodies (Cat# BNUM0200-50, Biotium, Fremont, CA, USA) (1:400 dilution in 100 mM carbonate-bicarbonate buffer, pH = 9.5) were coated onto a high binding 96-well plate (REF# 9018, Corning), and incubated with neutralized reaction for 1–2 h at room temperature (RT). After washing five times with 300 μL/well of wash buffer (phosphate-buffered saline (PBS), 0.05% Tween-20, pH = 7.4), 100 μL/well of Europium (Eu)-labelled streptavidin (Cat# 1244–360, PerkinElmer, Waltham, MA, USA) (1:1000 dilution in PBS, 1% BSA, pH = 7.4) was added and incubated for 1 h at RT. After washing 5 times again with 300 μL/well of wash buffer, DELFIA^®^ time-resolved fluorescence (TRF) intensity was detected using a Tecan Infinite^®^ M1000 after adding 100 μL/well of enhancement solution (Cat# 1244–105, PerkinElmer). Non-specific binding was removed through extensive wash with wash buffer. Competitor peptide with Eu TRF intensity that was lower than 25% of Bio-EBV epitope alone was considered strong binder, while peptide with 25–50% was weak binder.

In silico binding: The DQ0602 peptide binding prediction algorithm (http://tools.iedb.org/mhcii/) was used to assess if mutated motifs derived from the mass spectroscopic readouts changed DQ0602 peptide-binding registers. Consequently, three 20 mer peptide variants derived from the X179 strain, wild type pH1N1, and mutated motif from the vaccines were used to query the DQ0602 binding prediction server [36]. The 20 mer peptide stretches surrounding the mutated motif (10 aa upstream and 10 aa downstream of the mutated motif) or the homologous position in the reference strains were constructed using custom python scripts. 

### 2.5. Tetramer Analysis

For a few selected peptides where mutations were found to alter T cell reactivity when presented by DQ0602 potentially, and when these could be hypothesized to explain why Pandemrix could have been more narcolepsy triggering than Arepanrix, DQ0602 peptide tetramers were created and tested in six narcolepsy cases (5 post Pandemrix, one recent onset) and 4 DQ0602 control cases (all vaccinated with Pandemrix in 2009–2010) as described in Luo et al. [32].

## 3. Results 

### 3.1. Characterization of Protein Content in Arepanrix and Pandemrix 

**Figure 1:**
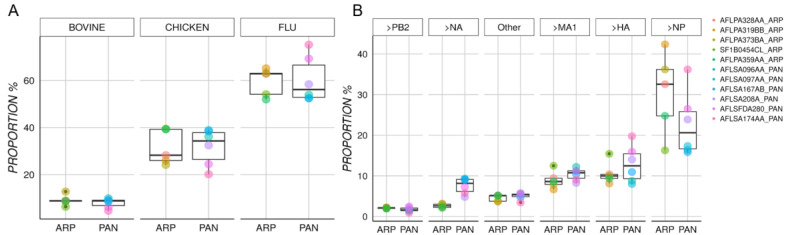
Protein composition in Pandemrix versus Arepanrix. (**A**) Boxplots for each type of protein, Table 1. (**B**) This figure shows the relative proportion or concentration of each protein type within each vaccine for peptide fragments classified as influenza proteins.

Each vaccine lot was digested with trypsin and chymotrypsin and then subjected to MS characterization, thereby ensuring high coverage of the representative protein content. Mean coverage for the influenza proteins was 80.5% in Pandemrix versus 71.1% in Arepanrix (Supplementary Figure S1). We observed highly similar mean global proportions of influenza (Pandemrix 60.2%; Arepanrix 59.4%), chicken (Pandemrix 31.7; Arepanrix 31.4%), and bovine proteins (Pandemrix 7.9%; Arepanrix 9.1%) in these vaccines (Figure 1A). Such results are consistent, given that both vaccines are produced from the same parent NYMC X-179A reassortant virus consisting of PR8 backbone and pH1N1 surface proteins. Bovine proteins are likely reflecting deoxycholate solubilization, as this compound is isolated from bovine gallbladder extracts.

In Pandemrix, influenza neuraminidase (NA) was significantly overrepresented (7.56% vs. 2.64%; *p* = 0.0012), hemagglutinin (HA) was moderately increased (12.9% vs. 10.6%, *p* = 0.3), while nucleoprotein (NP) was underrepresented (22.69% vs. 30.44% *p* = 0.2) (Figure 1B). The proportion of other influenza proteins such as matrix protein (MA1), polymerase PB2, polymerase PA, and polymerase PB1 were similar in both Pandemrix and Arepanrix (Table 2). Although the global proportion of non-viral proteins were similar in both Arepanrix and Pandemrix, we observed that chicken proteins such as ApoB, Vitellogenin-2, Glucose-6-phosphate isomerase, Ovalbumin, Ezrin, and Annexin A2 were significantly overrepresented (*p* < 0.05) in Arepanrix (Table 2). The bovine proteins such as tubulin alpha-1B were significantly overrepresented in Pandemrix, while Junction plakoglobin was significantly overrepresented in Arepanrix.

### 3.2. Differential Mutation Proportions among Arepanrix and Pandemrix

**Figure 2:**
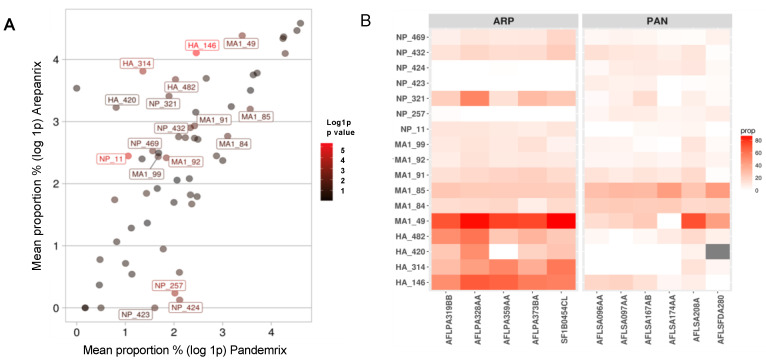
Mean mutation proportion in Arepanrix versus Pandemrix. (**A**) Each data point represents a specific mutated amino acid. This scatterplot maps the log-transformed mean mutation proportion in Pandemrix on the x-axis against log-transformed mean mutation proportion in Arepanrix on the y-axis. Calculated from a two-tailed Student’s t-test; the *p*-value of each data point indicates how significantly different the mutation is between vaccines. As data points are colored based on their *p*-value, the redder on the gradient scale, the more significant the mutation is in regard to differentiating Pandemrix and Arepanrix. (**B**) The heatmap is indicating the actual mutational proportion with the y-axis showing the mutated positions and the x-axis showing the vaccine lots.

A comparison of mutations in Pandemrix and Arepanrix uncovered 17 significantly interesting site-specific differences in relation to reference X-179A strain. 4 HA motifs were represented differently among the vaccines. Most significantly, the HA 146 (p.Asn146Asp) residue, which is close to a receptor binding site that interacts with human respiratory epithelial cells to initiate infection, was deamidated nearly six times more in Arepanrix (59.7%) than Pandemrix (10.7%). This mutation was by far the most significant difference among the two vaccines (*p* = 4.4 × 10^−6^), confirming previous observations that the difference in HA 146 was an apparent outlying mutation between Arepanrix and Pandemrix [30]. The other mutated residues in hemagglutinin included HA 314 (p.Pro314Gln, *p* = 5.7 × 10^−4^), HA 482 (p.Phe482Tyr, *p* = 8.4 × 10^−3^), and HA 420 (p.Arg420Ile, *p* = 4.2 × 10^−2^), all significantly enriched at least 3-fold in Arepanrix (Table 3, Figure 2A). 

NP, which is derived from PR8 and not pH1N1 sequence, also accumulated mutations that differentiated the two vaccines. For instance, the NP 11 (p.Glu11Gln, *p* = 2.8 × 10^−5^) residue mutated nearly 5 times more in Arepanrix (10.5%) than Pandemrix (1.9%), while deamidations were frequently enriched in Arepanrix at NP 321 (p.Asn321Asp, *p* = 0.02) and NP 432 (p.Asn432Asp, *p* = 0.01). It should be noted that some NP mutations occurred more frequently in Pandemrix than Arepanrix; the most significant of these include the following residues: NP 257 (p.Thr257Ala, *p* = 5.2 × 10^−4^), NP 423(p.Thr423Arg, *p* = 3.1× 10^−2^) and NP 424 (p.Thr424Ile, *p* = 3.6 × 10^−3^) (Table 3 and full list of mutations per batch in Supplementary Table S6). 

Matrix protein 1, also derived from PR8, was observed to have a differential accumulation of mutations between Arepanrix and Pandemrix. The most significant differences include the residue M1 49 (p.Arg49Ile, *p* = 3.9 × 10^−3^) as well as adjacent motifs deamidated at M1 91 (p.Asn91Asp, *p* = 1.6 × 10^−2^), M1 92 (p.Asn92Asp, *p* = 2.6 × 10^−3^) and M1 99 (p.Leu99Met, *p* = 2.1 × 10^−2^), that were all over represented in Arepanrix. Meanwhile, two adjacent motifs at M1 84 (p.Leu84Pro, *p* = 2.2 × 10^−2^) and M1 85 deamidation (p.Asn85Asp, *p* = 1.7 × 10^−2^) were enriched in Pandemrix (Table 3). 

Statistical comparison of mutation proportions revealed a general trend in increased differential mutations across in Arepanrix compared to Pandemrix and, as shown in Figure 2. Of interest, statistically significant (*p*-value < 0.05) mutations, such as HA 146, HA 314, and NP 11, typically occurred more often in Arepanrix than Pandemrix. 

### 3.3. DQ0602 Binding of Mutated Motifs in Arepanrix and Pandemrix

We next sought to determine whether these mutated and enriched motifs (Table 3) in either Pandemrix or Arepanrix modified their HLA binding registers and subsequently their overall propensity to bind narcolepsy associated DQ0602 allele. A combination of in-vitro and in-silico methods was adopted to address this question. We synthesized 15 mer peptide stretches overlapping by 4mers using the most abundant viral proteins (HA, NA, NP, M1 & PB2) from reference strains (X179A and wild type pH1N1) as a template. These 15 mer peptides were used to quantify DQ0602 binding affinities relative to a known EBV derived 15 mer peptide that is a strong binder [32]. In this way, a database of experimentally derived peptide binders from the abundant viral proteins was built. Next, we compared the mutated motifs (Table 3) from the vaccines to the reference motifs already tested for DQ0602 binding.

Among the mutations described in Table 3, six of the variant motifs changed the DQ0602 binding register. First, we confirmed that the previously described HA 146 (N to D) mutation enriched in Arepanrix changed peptide-binding register to bind strongly to DQ0602 (N allele 4.4%, D allele 53.47%). Second, we identified five novel mutations that changed DQ0602 binding registers. NP 257 (T to A) was enriched in Pandemrix and changed the binding register to DL[A]FLARSA (A allele 4.8%) from the reference DL[T]FLARSA (T allele 15.7%), this change is again projected to increase the binding affinity to DQ0602. Other mutations that modified DQ0602 binding registers are NP 423, HA 420, MA1 49 (see Supplementary Tables S1 and S2). Considering their differential abundance in Pandemrix versus Arepanrix and expected changes in binding register, we determined that only four mutations i.e., HA 146, NP 423, NP 424, and NP 257, have the potential to explain differential effects of these vaccines on narcolepsy.

### 3.4. Tetramer Studies of Four Mutated Motifs that Could Have Impacted Narcolepsy Risk

Our analysis projected that four Pandemrix enriched mutations (i.e., HA 146, NP 423, NP 424, and NP 257, see Supplementary Tables S1 and S2) within the DQ0602 binding registers could influence narcolepsy susceptibility. We thus conducted DQ0602 tetramer screens of the mutated motifs (Table S3) in expanded PBMCs as described previously [32] split into three conditions (i.e., stimulated with Arepanrix, Pandemrix, or specific mutated peptide) to identify cognate mutated peptide-specific CD4^+^ T cells. Six narcolepsy cases (5 post Pandemrix, one recent onset) and 4 DQ0602 control cases (all vaccinated with Pandemrix in 2009–2010) described in Luo et al. [32] were selected for this screen. While there appeared to be sporadic reactivity to some mutated motifs, we did not observe any significant differences in the frequencies of tetramer specific CD4 T cells in narcolepsy cases vs. controls (supplementary Tables S4 and S5). In comparison to immunodominant motifs we identified in our prior screens in these same patients, these epitopes [18] were considered insignificant.

## 4. Discussion

This study extends the Jacob et al. report [30] where only single batches of Arepanrix and Pandemrix were analyzed and presents a detailed characterization of the mutational burden and protein content of 5 Arepanrix and 6 Pandemrix batches. Mean coverage of the mass spectrometric characterization of influenza proteins, while still at high 71.1% in Arepanrix and 80.5% in Pandemrix, was slightly less than what Jacob et al. [30]. The sampled lots were actual vaccine doses used during the 2009 pandemic influenza vaccination campaign in Northern Europe and Canada, except for two lots that were monovalent bulks and sourced directly from GSK (see Table 1). Not surprisingly, considering that both Arepanrix and Pandemrix were derived from NYMC X-179A [26,30], the mean global proportions of Influenza, chicken and bovine proteins were comparable between the two vaccines (Figure 1). This finding agrees with Jacob et al. [30], where similar global proportions were observed. The four main influenza proteins in order of abundance were NP, HA, M1, NA, and PB2 (Table 2), which is consistent with other studie [26,30], while PB1, NS1 and nuclear export protein (NEP) were only present at low concentrations (<1%) in both the vaccines. 

We found that NA was significantly enriched three-fold in Pandemrix as compared to Arepanrix confirming the trend identified previously in Jacob et al. [30]. In contrast, NP was underrepresented in Pandemrix compared to Arepanrix, although this difference was not statistically significant. This finding conflicts with the reports of Jacob et al. [30], Vaarala et al. [33] and Ahmed et al. [34], all of which observed an overrepresentation of NP content in Pandemrix. The main limitation in these studies mentioned earlier is however that only one representative batch of each vaccine or the monovalent antigen bulk [30,33]. In addition, the protein content was characterized by various techniques including western blots, PAGE gels and mass spectrometry in these studies. In this study, we have characterized 6 different Pandemrix lots and 5 different Arepanrix lots, with the majority of them being actual vaccine doses. Further, using both trypsin and chymotrypsin protein digests to increased our protein coverage thus the finding may be more reliable. However, we did not perform any enrichment before MS characterization, which may have influenced our current results.

As recently reported, we found that vaccine strains, like wild type virion infecting hosts, mutate in culture, and this leads to divergences in vaccine viral sequences in different vaccines or across vaccine batches. Differences may thus depend on how often the manufacturer reuses the primary NYMC strain versus continuing to amplify isolates from their own egg cultures for future propagation. As an example, Skowronski et al. found that H3N2 reassortant vaccine strains had mutated in key antigenic residues, likely contributing to reduced efficacy in 2012–2013 [37]. Similarly, Jacob et al., conducting Mass Spectrometry (MS) characterization of X-179A derived pH1N1 vaccines, 2009 Pandemrix and 2010 Arepanrix, discovering that a specific HA mutation, N146D, had accumulated in Arepanrix, distinguishing the two antigens [30] (limitation in the Jacob et al. study was that only a representative batch of actual Pandemrix vaccine (batch DFLSA014A) and bulk Arepanrix (batch SF1B0454Cl) was studied and compared). Further, the Arepanrix lot was a lot that had never been used and had been prepared one year after the pandemic (2010). In this study, we could confirm the dominance of N146D in Arepanrix but not Pandemrix across all lots. Interestingly, this mutation conferred higher growth and was selected in subsequent pH1N1 strains X-181 [26]. It may thus be that the mutation accumulated in Arepanrix but not Pandemrix because of differences in Arepanrix culture procedures.

As it was conceivable that the N146 sequence found in Pandemrix and wild type H1N1 but not Arepanrix was essential to explain narcolepsy susceptibility, we further examined binding of both N146 and D146 peptides on DQ0602 molecules, confirming in vitro prediction indicating that 146 binds with lower affinity to DQ0602, another factor that could contribute to different susceptibility. Using DQ0602 tetramers for sequences; however, we found that very few T cells recognized these peptide sequences in both narcolepsy and control subjects, making it unlikely to be of significance in narcolepsy pathophysiology. Similar to the study of HA N146D, we also studied tetramers for NP T424I, NP T423I, and NP T257A, three other mutations that are much more abundant in Pandemrix versus Arepanrix (Table 1) and were predicted to bind DQ0602 with an equivalent affinity (supplementary Table S1). In vitro binding studies indeed found that all these peptides bound DQ0602 with high affinity. However, tetramer studies in narcolepsy and controls did not support abundance for T cells recognizing these epitopes making it unlikely to be functionally important.

With recent results suggesting that a potential mimic of HCRT_NH2_ is pHA_273–287_, we also carefully examined frequency and sequence variation within this segment, present in both wild type pH1N1, X-179A and X181, but could not find any mutation or difference in frequency across vaccines, making it unlikely composition difference at this level explain differential narcolepsy risk. It is nonetheless interesting to note that pHA_273–287_ contains N at position 273 (predicted to bind DQ0602 in P1) and that this residue is partially glycosylated [30], a modification that could make a difference in B cell reactivity and perhaps epitope processing and presentation to T cells. A future direction would be to profile in detail the post-translational modifications in these motifs across the two vaccines. For instance, possible deamidation in the MHC binding pocket could alter the DQ0602 binding register and glycosylation in or flanking the pHA_273–287_ or in other cases, PTMs such as N-linked or O-linked glycosylation have also been shown to protect epitope cleavage sites, prevent efficient antigen processing, or influence recognition by cognate T-cell receptors [38]. Glycosylation patterns in key residues across vaccines are, therefore, also of interest in the context of DQ0602 binding and narcolepsy susceptibility. 

## 5. Conclusions

In summary, characterization of the mutation load of several Pandemrix and Arepanrix lots revealed extensive differences in influenza mutation frequencies when compared across the vaccines and also in relation to the parent vaccine strain X179A. We also did not find any single mutations within the most likely culprit mimic sequence that may trigger autoimmunity, pHA_273–287_ in this study. Future research exploring double mutations or PTMS in this region (pHA273–287) and additional flanking regions are underway in our laboratory and could yield additional answers. Nonetheless, as identified in this study, the vaccine composition is complex and diverse, and thus unrecognized differences could still play a role. As an example, it is notable that a significant portion of spectrograms in these vaccines does not map to any known protein sequence, thus our search for differences that could be involved in narcolepsy triggering was in no way exhaustive. Beside vaccine differences, it may also well be that difference in past or concomitant microbial exposure of the Canadian (vaccinated with Arepanrix), and northern European population (vaccinated with Pandemrix) could be primarily involved, for example, timing of concomitant pH1N1 infection, past H3N2 infections, or presence of specific streptococcal A infections, as suggested by other studies [39].

## Figures and Tables

**Table 1:** Characteristics of Pandemrix and Arepanrix lots used in the Mass Spectrometry (MS) characterization.

Vaccine	Batch	HA (ug/mL)	Type	Origin	Viral Strain
Arepanrix	AFLPA328AA	15	Vaccine	Canada	X179A
Arepanrix	AFLPA359AA	15	Vaccine	Canada	X179A
Arepanrix	AFLPA373BA	15	Vaccine	Canada	X179A
Arepanrix	AFLPA319BB	15	Vaccine	Canada	X179A
Arepanrix	SF1B0454CL	457	Bulk	GSK	X179A
Pandemrix	AFLSA208A	15	Vaccine	GSK	X179A
Pandemrix	AFLSA174AA	15	Vaccine	France	X179A
Pandemrix	AFLSFDA280	139	Bulk	GSK	X179A
Pandemrix	AFLSA167AB	15	Vaccine	Sweden	X179A
Pandemrix	AFLSA097AA	15	Vaccine	Sweden	X179A
Pandemrix	AFLSA096AA	15	Vaccine	Sweden	X179A

**Table 2:** The protein content of Arepanrix and Pandemrix.

Protein	Organism	Pandemrix	Arepanrix	CI95 U ± L	Statistic	*p*-Value
Nucleoprotein NP	PR8	22.69% (1600)	30.44% (1092)	20.8 ± −5.3	1.389	0.2044
Hemagglutinin HA	pH1N1	12.9% (894)	10.66% (420)	2.9 ± −7.3	−1.003	0.3436
Matrix Protein 1 MA1	PR8	10.39% (724)	9.01% (357)	1.4 ± −4.1	−1.197	0.2709
Neuraminidase NA	pH1N1	7.56% (533)	2.64% (99)	−2.9 ± −7	−5.997	0.0012
Polymerase PB2	PR8	1.7% (109)	2.09% (80)	1 ± −0.2	1.655	0.1528
Polymerase PA	pH1N1	1.81% (124)	1.34% (56)	0.5 ± −1.5	−1.12	0.3003
Polymerase PB1	pH1N1	1.4% (91)	1.33% (52)	0.6 ± −0.7	−0.266	0.7985
Nonstructural protein 1	PR8	0.52% (34)	0.45% (18)	0.1 ± −0.3	−0.821	0.4404
Nuclear export protein	PR8	0.11% (10)	0.26% (11)	0.4 ± −0.1	1.605	0.1496
Apolipoprotein B	*Gallus gallus*	0.16% (11)	2.07% (87)	3.2 ± 0.6	3.965	0.016
Vitellogenin−2	*Gallus gallus*	0.12% (8)	1.15% (47)	1.5 ± 0.6	5.772	0.0024
Glucose−6−phosphate isomerase	*Gallus gallus*	0.49% (35)	0.87% (31)	0.7 ± 0	2.684	0.0442
Ovalbumin	*Gallus gallus*	0.39% (28)	0.85% (33)	0.8 ± 0.1	2.872	0.02
Annexin A2	*Gallus gallus*	0.55% (37)	0.73% (28)	0.4 ± 0	2.382	0.0434
Ezrin	*Gallus gallus*	0.58% (40)	0.73% (28)	0.3 ± 0	2.754	0.0246
Junction plakoglobin	*Bos taurus*	0.15% (9)	0.69% (23)	1.1 ± 0	2.586	0.0496
Tubulin alpha−1B chain	*Bos taurus*	0.88% (60)	0.54% (21)	−0.1 ± −0.6	−3.212	0.0147

**Table 3:** Significantly different mutated motifs as compared to X179A strain in six batches of Pandemrix^®^ and five batches of Arepanrix^®^.

Protein Pos (Intial > Mut)	*p*-Value	T−Stat (CI U ± L)	Mean Proportion%	
			Arepanrix	Pandemrix
HA 146 (N > D)	4.40 × 10^−6^	10.2 (60 ± 38)	59.7	10.7
HA 314 (P > Q)	5.70 × 10^−4^	7.3 (55.6 ± 27)	44.2	2.9
HA 420 (R > I)	4.42 × 10^−2^	2.8 (45 ± 1)	24.2	1.3
HA 482 (F > Y)	8.24 × 10^−3^	4.3 (51.2 ± 12.6)	38.5	6.6
M1 49 (R > I)	3.90 × 10^−3^	4.6 (76.8 ± 23.4)	79.1	29
M1 84 (L > P)	2.24 × 10^−2^	−2.9 (−1.2 ± −11.6)	14.9	21.3
M1 85 (N > D)	1.78 × 10^−2^	−3.4 (−2.8 ± −19)	23.5	34.4
M1 91 (N > D)	1.66 × 10^−2^	3.1 (13.2 ± 1.8)	17.7	10.3
M1 92 (N > D)	2.61 × 10^−3^	4.2 (7.6 ± 2.2)	10.2	5.3
M1 99 (L > M)	2.12 × 10^−2^	2.8 (11 ± 1.1)	10.4	4.3
NP 11 (E > Q)	2.80 × 10^−5^	7.9 (11.1 ± 6.2)	10.5	1.9
NP 257 (T > A)	5.20 × 10^−4^	−6.8 (−4 ± −8.5)	0.3	6.5
NP 321 (N > D)	2.14 × 10^−2^	3.3 (41.8 ± 5.3)	29.2	5.7
NP 423 (T > R)	3.19 × 10^−2^	−2.9 (−0.5 ± −7.4)	0	4
NP 424 (T > I)	3.65 × 10^−3^	−5.1 (−3.6 ± −10.8)	0.1	7.3
NP 432 (N > D)	1.28 × 10^−2^	3.1 (13.6 ± 2.1)	17.2	9.3
NP 469 (E > D)	1.13 × 10^−2^	3.9 (12.9 ± 2.6)	11.5	3.8

## Supplementary Materials

The following are available online at https://www.mdpi.com/2076-393X/8/4/630/s1, Figure S1: The percentage coverage of the main influenza viral proteins as characterized by mass spectrometry across different vaccine lots derived from Pandemrix and Arepanrix post digestion with trypsin and chymotrypsin. Table S1: Frequency of mutation in Arepanrix, Pandemrix and native sequence in pH1N1 2009, with predicted DQ0602 binding affinity, binding repertoire and possible narcolepsy effect, Table S2: Frequency of potentially narcolepsy- predisposing mutation in Arepanrix, Pandemrix and native sequence in pH1N1 2009, with actual in vitro DQ0602 binding effects, binding repertoire and tetramer results, Table S3: Peptide motifs selected for additional tetramer screening, Table S4: Mean Frequencies and logistic regression of Mutated Peptide tetramer specific CD4 Tcells in narcolepsy cases and controls after stimulation with pandemrix, arepanrix or no stimulation, Table S5: Frequency of Mutated Peptide tetramer specific CD4 Tcells in narcolepsy cases and controls after stimulation with pandemrix, arepanrix or no stimulation, Table S6: All mutated occurences and unmutated occurences extracted from the mapped spectra for all vaccines and lots (Nan—indicated not found).
