# Supplementary material for: Mass Spectrometric Characterization of Narcolepsy-Associated Pandemic 2009 Influenza Vaccines
Source: Vaccines (Basel). 2020 Oct 30;8(4):630. doi: 10.3390/vaccines8040630 (PMC7712488; doi:10.3390/vaccines8040630)
Supplement: Supplementary file 1 [file vaccines-08-00630-s001.zip › vaccines-942115 sup/vaccines-942115 XML sup .pdf]

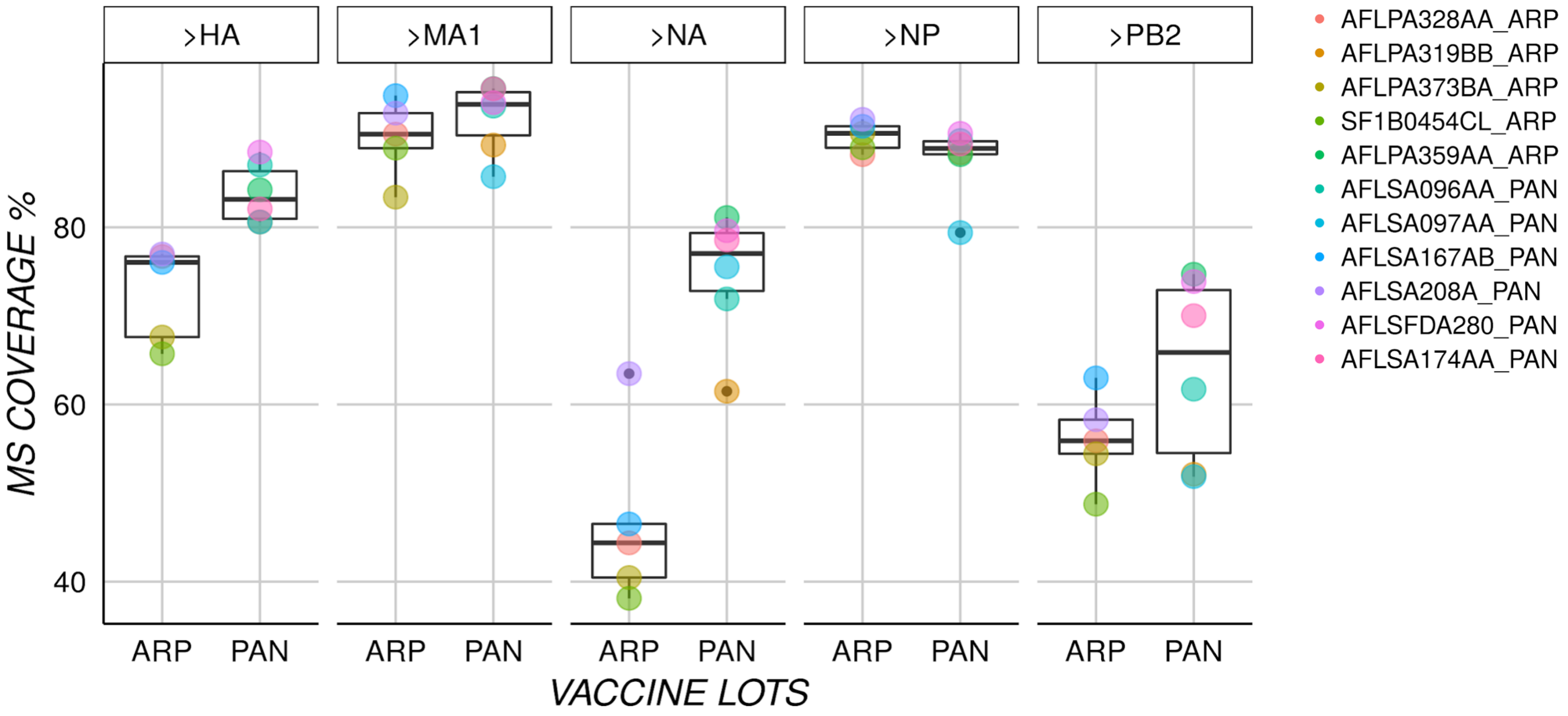

Supplementary Figure 1: The percentage coverage of the main influenza viral proteins as characterized by mass spectrometry across different vaccine lots derived from Pandemrix and Arepanrix post digestion with trypsin and chymotrypsin.
